# Healthy Aging from the Perspectives of 683 Older People with Multiple Sclerosis

**DOI:** 10.1155/2016/1845720

**Published:** 2016-07-18

**Authors:** Elizabeth M. Wallack, Hailey D. Wiseman, Michelle Ploughman

**Affiliations:** Recovery & Performance Laboratory, Faculty of Medicine, Memorial University, St. John's, NL, Canada A1A 1E5

## Abstract

*Purpose.* The aim of this study was to determine what factors most greatly contributed to healthy aging with multiple sclerosis (MS) from the perspective of a large sample of older people with MS.* Design and Methods.* Participants (*n* = 683; >55 years of age with symptoms >20 years) provided answers to an open-ended question regarding healthy aging and were categorized into three groups, 55–64 (young), 65–74 (middle), and 75 and over (oldest old). Sociodemographic actors were compared using ANOVA. Two independent raters used the framework method of analyzing qualitative data.* Results.* Participants averaged 64 years of age (±6.2) with MS symptoms for 32.9 years (±9.4). 531 participants were female (78%). The majority of participants lived in their own home (*n* = 657) with a spouse or partner (*n* = 483). Participants described seven themes: social connections, attitude and outlook on life, lifestyle choices and habits, health care system, spirituality and religion, independence, and finances. These themes had two shared characteristics, multidimensionality and interdependence.* Implications.* Learning from the experiences of older adults with MS can help young and middle aged people with MS plan to age in their own homes and communities. Our data suggests that older people with MS prioritize factors that are modifiable through targeted self-management strategies.

## 1. Purpose

Multiple sclerosis (MS) is an autoimmune disease characterized by demyelinating lesions and atrophy of the central nervous system [1]. Varying severity and progression of a wide range of sensorimotor and cognitive symptoms make it highly heterogeneous [1]. MS is most often diagnosed between the ages of 18 and 40 years and affects women more than men [2]. According to the World Health Organization, there are 2.3 million people living with MS worldwide. Canada has the highest prevalence of MS with 291 per 100,000 [3]. The number of people with MS who are older adults is increasing, likely due to improved longevity and more effective treatments [4]. Recent evidence also suggests that the disease course may be modifiable by engaging in healthy lifestyle behaviors such as exercise, eating fish, abstaining from smoking, and moderate alcohol consumption [5, 6]. Furthermore, the presence of cardiovascular comorbid conditions, which are behaviorally driven, is associated with hastened disease progression [7].

Healthy aging with MS is a relatively new concept [8, 9] and whether healthy aging with MS differs from generic models of healthy aging is not known. Finlayson and group's early research among older people with MS aligned with Rowe and Kahn's (1997) health-based “Successful Aging” model which focused on absence of disability and disease, high cognitive and physical capacity, and active engagement with life. They found that older people with MS reported having less freedom and needed more assistance than their friends and family who did not have MS [10, 11]. Later, along with and Dilorenzo et al. (2008), Finlayson and colleagues suggested that older people with MS identified social resources and made adaptations to living with MS [10, 12]. This fits with the Selective Optimization with Compensation (SOC) model of healthy aging, which focuses on strengths rather than deficits, suggesting that older people select and optimize their best abilities and most intact functions while compensating for declines and losses [13].

There is further evidence to suggest that the SOC model is relevant to those who age with a chronic illness. Kralik [14] suggested that people with a chronic illness can learn to live with their condition, integrating it into their daily lives. Individuals can gain extensive knowledge about their illness and, in the process, develop skills in self-management [9, 15]. Young et al. [16] suggested that successful aging may coexist with diseases and functional limitations if compensatory psychological and/or social mechanisms are employed.

A new multitiered model of healthy aging with MS was proposed by Ploughman and colleagues in 2012 [8]. Work, social engagement, effective and accessible health care, healthy lifestyle habits, and maintaining independence at home were found to be critical proximal factors of healthy aging [8]. Financial flexibility, social support, cognitive and mental health, and resilience provided supporting foundational factors [8]. Thus far, the studies in this field have sampled small groups of participants [2, 8, 10–12]. Whether models of healthy aging proposed by previous research fit with a large diverse sample of older people with MS is not known.

The aim of this study was to determine, for the first time, what factors most greatly contributed to healthy aging with MS from the perspective of a large sample of older people living with MS. We hypothesized that factors influencing healthy aging among the young old (55–64) would differ from those identified by those aged 65–74 and the oldest old group (>75).

## 2. Design and Methods

### 2.1. Participants

After receiving ethical approval from the Health Research Ethics Authority, we accessed secondary data from a dataset of older adults with MS. Following pretesting of survey questions relating to health, lifestyle, and aging with MS [8], the original survey information was obtained by mail or telephone and was entirely self-reported. To be included in the survey, participants were 55 years of age and older and living with MS symptoms for 20+ years. On the last page of the survey, participants were asked to respond to the question, “*From your point of view, what are the most important things that help you live long and healthy with MS?*”

### 2.2. Analysis

#### 2.2.1. Quantitative Analysis

To compare age categories, participants were divided into three groups, 55–64 (young), 65–74 (middle), and 75 and over (oldest old). We also extracted variables from the original dataset to help characterize the age groups. Mental health status was measured using the Hospital Anxiety and Depression Scale (HADS). Scores for the scale (emotional distress) ranged from 0 to 42, with higher scores indicating more distress [17]. Level of disability was measured using the Barthel Index where a score of 100 indicates total independence and a score of 0 indicates total dependence [18]. Financial situation was described as either (a) “I have more than enough money to meet my needs so I can live the way I want,” (b) “I have enough money to meet my needs so I can live the way I want,” or (c) “I do not have enough money to meet my needs so I can live the way I want.” Living situation was described as (a) “I live alone,” (b) “I live with my partner,” (c) “I live with my children,” or (d) “I live with a friend or extended family.” Type of home was described as (a) “I live in a house/apartment/condominium,” (b) “I live in an assisted living complex,” or (c) “I live in a long-term care facility.” A one-way between-subjects ANOVA was conducted using SPSS 22.0 to compare the effect of age on each of the five variables described above.

### 2.3. Qualitative Analysis

The response text from the target survey question was imported into QSR NVivo 10 software (NVivo) for analysis. Two independent raters (EW and HW) used the framework method of analyzing qualitative data described by Gale et al. [19] which follows seven stages. Because this was secondary analysis, stage 1, transcription, was not necessary. The transcription process used by Ploughman et al. (2014) is described elsewhere [2]. In the next stage, familiarization required both raters to read and reread responses from participants recording initial thoughts and impressions. During stage 3 the researchers coded the first 100 responses by reading each line of text independently and created categories that described what was important about each passage. Stage 4 involved developing a working analytical framework and coders met to compare the labels that were applied to the categories. Some codes were grouped together and some coding names were changed to reflect a shared perspective on the category. This step also provided opportunities to discuss disagreements between coders and negotiate resolutions. Coders rearranged previously coded responses to match newly developed shared coding names and some comments were placed into multiple categories by one or both raters to better reflect the complexities of responses. This formed the working analytical framework that was used to code the next 100 responses.

Coders alternated between stage 3 and stage 4 every 100 responses, modifying the emerging framework at each step until no further modifications were required. Each coder kept a journal of changes that were made. The majority of disagreement occurred within the first 300 responses. The 5th stage required the coders to independently index all remaining responses using the existing categories and codes. Stage 6 involved summarizing the data into the framework matrix. One coder created subcategories in NVivo and identified quotations that illustrated the concepts. In the final stage the researchers compared the newly built framework to those described by others [8, 13, 20]. We used the number of times a theme/subtheme was referenced (numerator) as a proportion of the total number of references for all themes/subthemes (denominator) to create summary figures. Pseudonyms were used when citing quotations from participants.

To determine intercoder agreement, we performed a two-way mixed, consistency, average-measures Intraclass Correlation (ICC) using SPSS version 22.0. High ICC values indicate greater interrater reliability (IRR), with an ICC estimate of 1 indicating perfect agreement and 0 indicating only random agreement [21]. Negative ICC estimates indicate systematic disagreement. We used cutoffs for qualitative ratings of agreement based on ICC values described by Cicchetti (1994), where IRR is poor for ICC values less than 0.40, fair for values between 0.40 and 0.59, good for values between 0.60 and 0.74, and excellent for values between 0.75 and 1.0 [22].

## 3. Results

### 3.1. Participants

Of 921 surveys, 743 were returned [2]. The final sample consisted of 683 people aged within 55–88 years (M = 64, SD = 6.2) who responded to the open-ended question. The participants' years since onset of MS symptoms averaged 32.9 years (SD = 9.4). Five hundred and thirty-one of the participants were female (78%). The majority of participants lived in their own home (*n* = 657) with a spouse or partner (*n* = 483) and required some assistance for activities of daily living (Barthel Index, Table 1). There were no significant differences between the age groups with respect to mental health status, financial situation, level of disability, type of home, or living situation. As expected there was a significant effect of age on years since onset of MS symptoms [*F*(2,679) = 32.56, *p* < 0.0001] (Table 1).

**Figure 1:**
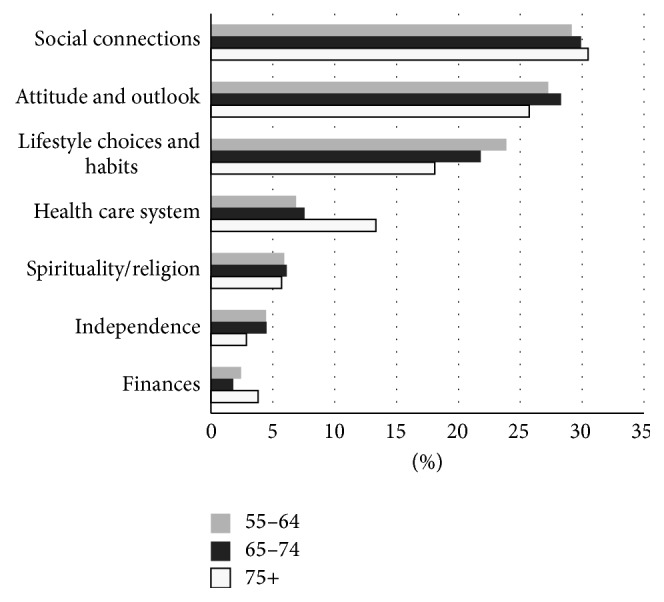
Seven determinants of healthy aging with MS. The average frequency of each theme by age category.

In terms of the qualitative results, the ICC of coded responses was in the excellent range, ICC = 0.999, with a 95% confidence interval of (0.995, 1.000) [22], indicating that coders had a high degree of agreement. The length of responses ranged from one word to 1,022 words. In total there were 1,403 coded items which represented responses from participants. Participants described seven overarching themes that contributed to healthy aging with MS from their points of view. These included (beginning with the theme with highest frequency) social connections, attitude and outlook on life, lifestyle choices and habits, health care system, spirituality and religion, independence, and finances (Figure 1). Participants' responses ranged from identifying a single theme to 6 out of 7 of the themes in one response. These seven themes had two shared characteristics, multidimensionality and interdependence.

### 3.2. Seven Determinants of Healthy Aging with MS

#### 3.2.1. Social Connections

“Social connections” was the most commonly identified factor contributing to healthy aging with MS. Descriptions of social connectedness accounted for 29% of the entire data set (Figure 1). Three types of social connections were identified: (1) reciprocal relationships with family and friends, (2) social engagement and volunteerism, and (3) support provider/recipient relationships. Older persons with MS valued social connections with family, friends, spouses/partners, children, grandchildren, neighbours, and even pets. Activities outside the home that provided opportunities for social engagement were also highly valued and included support groups, volunteer work, and community organizations.

Older people with MS were also contributing to their social networks. These interactions were often reciprocal in nature. One participant explained the following.

* My volunteer work with my dog and the hospital (patient advocacy) has kept me stimulated*. (Hélène, 59, Quebec)

Social connections were sometimes (but not always) structured in a support provider-care recipient dynamic, in which social networks provided emotional and instrumental support for older people with MS. These networks provided encouragement, opportunities for activity (both mental and physical), and help with activities of daily living. This is illustrated by the following example.

*People close to me who accept my MS and give me all the encouragement and support that I need, plus allow me to do the activities that I wish to do and at my level and speed*. (Isabel, 66, Nova Scotia)

Social connections also provided older people with MS a sense of purpose and helped to motivate them to be well. This was most evident through peoples' descriptions of their relationships with their children and grandchildren. One person explained the following.

* I wanted to see my kids go to university and get married and have children of their own*. (Marie, 62, Nova Scotia)

#### 3.2.2. Attitude and Outlook

**Figure 2:**
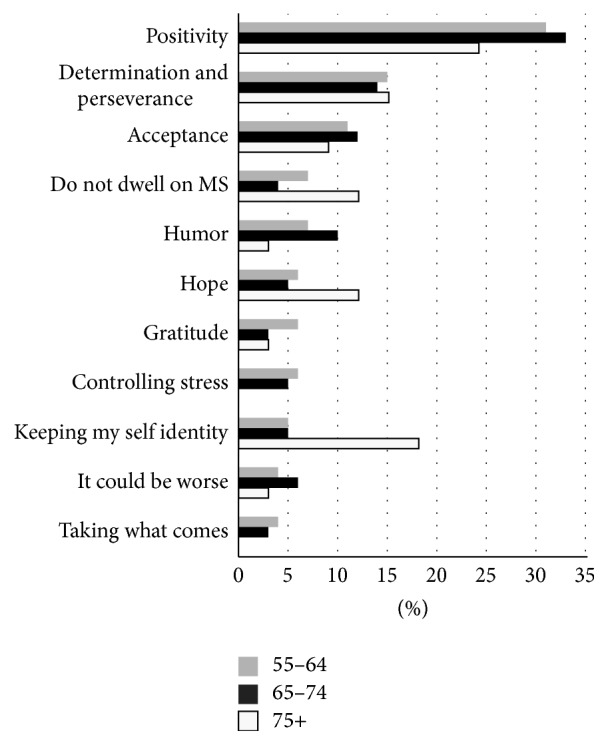
Age stratification of attitude and outlook subthemes. The figure displays the average frequency of each subtheme by age category. “Keeping my self-identity” is notably more prominent among those aged 75 and older.

Attitude and outlook was the second most commonly identified factor important to healthy aging with MS. This theme accounted for 27% of coded responses. Older people with MS reported adopting ways of thinking that helped them to cope with the challenges of living with MS. Among the 11 subthemes identified within this theme (Figure 2), the role of positive thinking was most commonly reported. Older people with MS described adopting a positive attitude and being optimistic. Second to positivity was determination and perseverance, two terms that were used interchangeably by participants. One example of this is as follows:

*Being determined has helped – I should have stopped work (teacher grade 1-3) five years before I did, perseverance*. (Michael, 64, Ontario)

Participants also placed importance on acceptance and described understanding how to work within their limitations and to focus on their abilities. Humor, being able to laugh, and looking for humor in difficult situations were also described as being important to older people with MS within this theme. Maintaining one's self identity was also highly valued among older people with MS. People frequently explained that “I am not my disease.” One participant described this saying the following.

*Life is what it is, I am what I am, life is good*. (Scott, 76, Nova Scotia)

#### 3.2.3. Lifestyle Choices and Habits

**Figure 3:**
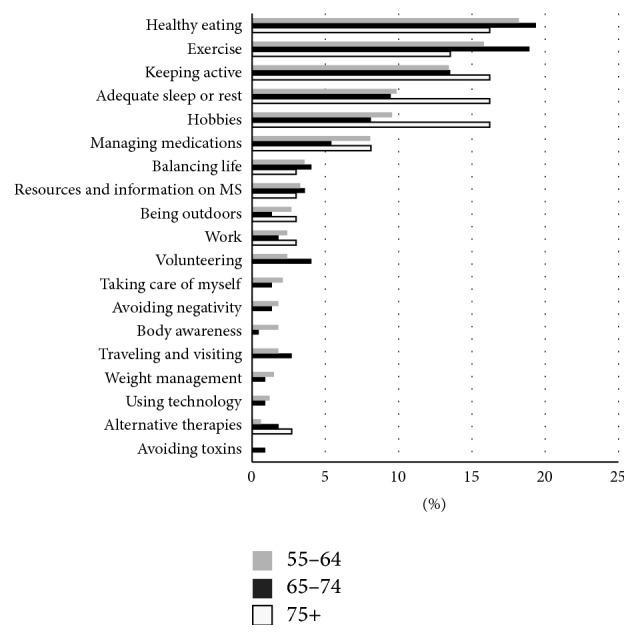
Age stratification of healthy lifestyle choices and habits. The figure displays the relative importance of each subtheme by age category. Those aged 75 and older identified just over half as many subthemes as the younger age groups.

This theme accounted for 23% of coded responses and had the largest number of subthemes, helping to illustrate how this concept was highly variable and individualized (Figure 3). Because of the large variety in this theme, we propose that lifestyle choices and habits can be understood by considering their relationship to (1) body or (2) purpose. Lifestyle choices that affect the body included healthy eating, exercise, keeping active, adequate sleep or rest, managing medications, alternative therapies, weight management, body awareness, and taking care of oneself. Those activities contributing to the participant's sense of purpose included hobbies, work, finding resources, and information about MS, being outdoors, choosing to balance life's demands, using technology, and traveling and visiting. Lifestyle choices and habits in the “body” subgroup potentially impacts an individual's physical health, whereas lifestyle choices and habits in the “purpose” subgroup relate to how an individual finds meaning in their lives and their mental health.

Another important characteristic of this theme was the way in which some individuals used very clear and specific terms to describe their lifestyle choices and habits, while others used more general descriptions. The following two quotations help to illustrate this characteristic.

*Keeping as active as possible and staying [in] a healthy lifestyle*. (Madonna, 56, Newfoundland)

*I have a healthy diet and get lots of rest. This year I am able to take Wednesdays as a sick day. This is extremely helpful. I can't walk because I don't have much strength in my legs. I get exercise on my exercise bike. I do 20 minutes a day. It really helps me feel better. I do not eat red meat*. (Karen, 57, New Brunswick)

#### 3.2.4. Health Care System

The health care system was the fourth most commonly described factor that contributed to healthy aging with MS, accounting for 10% of responses, considerably less than the top three themes (Figure 1). Within this theme, having access to high quality care was extremely important to respondents. People described high quality care as care that was prompt, reflexive, and appropriate. Older people with MS also highly valued relationships with their care providers. Participants valued health care professionals that listened to their opinions, acknowledged their feelings, and gave them encouragement. A wide range of health care professionals were cited by participants as being important within their circle of care including neurologists, professional caregivers, physiotherapists, and massage therapists.

The health care system theme also contained negative statements. These comments described frustration with lack of access to treatment alternatives including the Zamboni/liberation treatment and negative interactions with health care professionals.

#### 3.2.5. Spirituality and Religion

Six percent of the total comments in our sample described how spirituality and religion contributed to healthy aging with MS (Figure 1). Older persons with MS described how faith and belief in God helped them cope with the challenges of living with MS. Organized religion was found to be source of social support as well as an opportunity to help others through volunteer work. Some participants described spirituality in more general terms and cited activities such as prayer and meditation as being an important aspect of their lives.

#### 3.2.6. Independence

This theme only accounted for 3% of all responses (Figure 1). Older people with MS cited the importance of making adaptations to allow them to remain independent. These included using home health devices, wheelchairs, accessible vans, and making modifications to their homes. Participants also described how their living situation allowed them to remain independent, for example, living near their families and residing in an accessible home. Independence was also described as a personal choice or a way of thinking that overlapped with the theme attitude and outlook on life. This is illustrated by the following example.

*I try to do things myself, if I can't do it then I seek out help. I don't listen to people who tell me I can't do something*. (Maria, 61, British Columbia)

#### 3.2.7. Finances

While finances only accounted for 2% of responses (Figure 1), there was still considerable variety within this theme. People described the importance of receiving government funding for equipment and services, the role of pensions, long-term disability, and insurance for providing financial security. Some people described the importance of working and providing for their families. Older people with MS described how adequate finances allowed them to make choices about their health care, manage medications, remain in their homes with supports, and have the freedom to participate in activities that they enjoyed. There were also negative statements within this theme. Some individuals expressed concerns over the lack of funding for medications and treatment options and not being able to get insurance coverage because of their illness.

### 3.3. Shared Characteristics across Themes 

#### 3.3.1. Multidimensionality

We found that most of the participants attributed healthy aging with MS to multiple factors rather than focusing on a single strategy or resource. This characteristic was true within the themes. People reported drawing on multiple resources within a construct. For example, friends, family, and volunteer work were combined in order to meet people's need for support and opportunities to engage with others.

This characteristic of multidimensionality was also true across themes. Respondents selected different strategies in combination, drawing on what resources were available to them. For example, while some older people with MS drew on resources from within the health care system, their social networks, and made choices to promote a healthy lifestyle, others looked to spirituality, financial security, and home adaptations as strategies for healthy aging.

#### 3.3.2. Interdependence

In addition to being multidimensional, we found that strategies people employed were also interconnected. That is to say, the presence of one factor, such as social connections, often contributed to another, such as attitude and outlook. To illustrate this further we present the following example.

*My husband – he is amazing! He helps me maintain a positive attitude. I like to tell people to have ADD. My ADD is: attitude, determination, and destiny. I want to be an excellent example for my family and friends*. (Eleonore, 64, Nova Scotia)

This example illustrates the way in which strategies for healthy aging with MS impact one another. This can be seen as something positive because having strong resources in one area may help to build resources in another area. However, the reverse may also be true: if resources in one domain begin to deteriorate, this may impact other domains, suggesting that strategies for healthy aging can be either strong or fragile depending on what types of connections are present.

### 3.4. Determinants of Healthy Aging Differ Little between Age Groups

Age stratification revealed subtle differences between the three age groups (Figures 1, 2, and 3). As illustrated in Figure 1, among the oldest old, finances, social connections, and the health care system were more frequently reported than in the two younger age groups; however all three age groups reported that social connections, attitude and outlook, and lifestyle choices and habits were the three most important elements to living long and healthy with MS. No differences were found among the subthemes of social connections apart from some minor life course changes (i.e., support from parents disappeared among the oldest group (the role of friends also diminished)).

Within the attitude and outlook subthemes, the role of “keeping my self-identity” became much more important to the oldest old. This subtheme ranked as the 2nd most important element of attitude and outlook among this group as illustrated in Figure 2.

By examining the subthemes by age group within the lifestyle choices and habits theme, we also found differences as illustrated in Figure 3. The role of adequate sleep and rest and participation in hobbies became much more pronounced among the oldest old. This group also did not identify lifestyle choices and habits such as avoiding toxins, body awareness, and technology. However, all three age groups reported the same top 5 lifestyle choices and habits: healthy eating, exercise, keeping active, adequate sleep or rest, and hobbies. The oldest old demonstrated less variety in the strategies employed and reported fewer overall choices and habits; however the remaining strategies still retained a balance between body and purpose.

### 3.5. Implications

As young and middle aged people with MS plan to age in their own homes and communities and prepare for changes that come with living as an older adult with a chronic illness, self-management strategies targeting people with MS are paramount. Our data suggests that older people with MS clearly prioritized specific factors which contributed to healthy aging. The ways in which different factors interacted with one another were complex and individualized, supporting previous research suggesting older people living with a chronic disease can develop expertise in managing their own health and well-being [9, 15]. The oldest old's responses differed in subtle but important ways from the younger age groups in our data. Together, these findings help to further our understanding of what it takes to be healthy while aging with MS.

### 3.6. The Big Three: A New Model of Healthy Aging with MS

We found that the vast majority of responses from older people with MS (79%) attribute healthy aging with MS to combinations of “The Big Three,” namely, social connections, attitude and outlook on life, and lifestyle choices and habits. This suggests that, despite different life stages, the experience of living with a chronic illness is a unifying factor for older people with MS.

**Figure 4:**
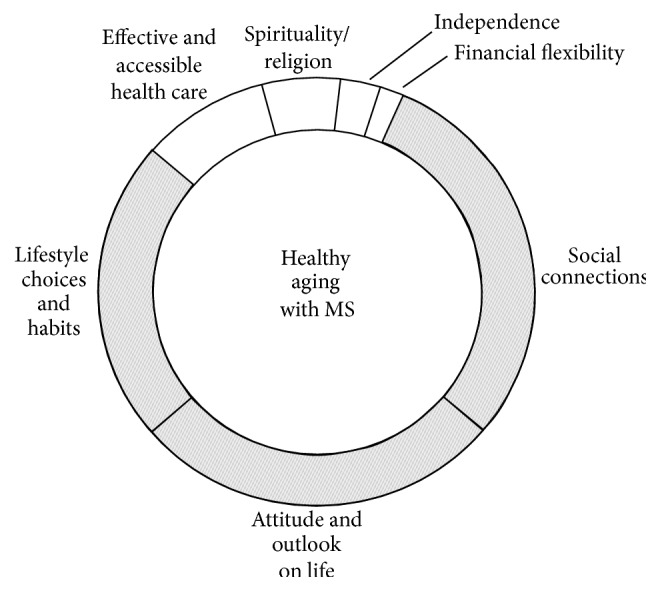
Healthy aging with MS. Healthy aging with MS is encircled by the seven contributing factors, illustrating the multidimensionality and interdependent nature of the connections. The model is comprised of two levels of factors: primary factors which are most important to older people with MS, which include social connections, attitude and outlook on life, and lifestyle choices and habits and secondary factors which are less prominently displayed in the model illustrating their relative importance and include effective and accessible health care, spirituality and religion, independence, and financial flexibility.

While previous research suggests psychosocial/SOC models of healthy aging with MS, our findings differ in two important ways. First, previous research identified the role of elements such as social support, attitude, spirituality, health care, and healthy lifestyle strategies in healthy aging with MS [8, 23–27]; however we suggest that older people with MS prioritize “The Big Three” over other elements (Figure 4). Next, the complex interconnections and multidimensional nature of the elements of healthy aging with MS suggest that a biopsychosocial model, which affects coping abilities as well as disease progression, may better reflect conceptualizations of healthy aging among older people with MS.

### 3.7. Lessons from the Oldest Old

Although our findings demonstrate only subtle differences between age groups, they may still offer important perspectives into what it takes to age successfully with MS. The oldest people in this sample (*n* = 51) provide unique insights because they have survived into old age (average 79 years of age) and successfully remained in their own homes and communities (40% living alone and 94% in their own home).

#### 3.7.1. Self-Identity

The oldest old placed higher value on the role of self-identity in healthy aging. Self-identity in older people with MS was previously identified as important to healthy aging [12]; however our findings describe its relative importance to the oldest old. Self-identity is part of an integration of experiences over time [28]. Some evidence suggests that older people with MS integrate experiences of living with illness into their identities [8] and positive self-identity has been linked to positive coping strategies, resilience, and persistence through adversity in older adults without a chronic illness [29–33]. People who adjust their goals to accommodate challenges are better able to preserve self-identity in the face of adversity [31]. Recently Skår and colleagues recommended that health care professionals acknowledge the importance of peer support for self-identity and empowerment in MS [34]. Future research in this area may wish to better understand the role of self-identity in people with MS and find ways to foster a positive sense of self among younger and middle age people with MS.

#### 3.7.2. A Shifting of Lifestyle Habits

The variety of healthy lifestyle choices and habits also narrowed among the oldest old. Some evidence suggests that there can be significant cultural and attitudinal differences between baby boomers (those aged 55–74 in our sample) and older generations [35]. This may explain why the oldest old in our study did not identify alternative approaches such as avoiding toxins, body awareness, and technology.

Activities can become more meaningful to an individual the longer and more frequently they are pursued [36]. This conceptualization follows the SOC model of healthy aging: as people age with MS they may choose to focus on a select few activities that are most beneficial and/or meaningful to them, devoting less time to less important activities.

Adopting certain healthy behaviors may also allow some individuals to survive into the oldest age category. There is evidence to suggest that the top 5 strategies identified by all the age groups (healthy eating, exercise, keeping active, adequate sleep/rest, and hobbies) do in fact lead to increased longevity in older adults without a chronic illness [37]. The oldest old may be those who chose to focus on the most beneficial lifestyle choices and habits throughout their lives. The fact that the oldest people with MS in our sample were able to pursue the same healthy lifestyle choices and habits as the younger groups suggests that these individuals may have overcome challenges that cause some people to stop participating in activities they once enjoyed, or that they live in environments that support healthy behaviors.

### 3.8. Self-Management Strategies for Healthy Aging with MS

#### 3.8.1. Fostering a Variety of Social Connections

Self-management interventions for people with MS focusing on the role of enhancing social connections are limited [38–41]. Our data supports that those interested in helping people with MS through self-management interventions should use inclusive language (moving away from language exclusively employing the support provider-care recipient dynamic) and consider a broad range of social connections when aiming to help address issues of social resources and communication. Young and middle aged people with MS should consider taking steps to foster and preserve social connections that include intergenerational links and reciprocal relationships with friends, family, and the greater community.

#### 3.8.2. Maintaining a Positive Sense of Self and the Role of Positive Psychology

While previous research suggests that inpatient rehabilitation for people with MS may help to foster a positive sense of self [34] to the best of our knowledge there are no recommendations on how to address this issue among community dwelling people with MS. Self-management strategies targeting self-identity among individuals living with a chronic illness are limited [42–44]. Strategies targeting young and middle aged people with MS should seek to adopt tools to foster and maintain a positive sense of self through the life span. Furthermore, future research should seek to understand if those who survive into very old age with MS develop a positive sense of self over time or if this is a preexisting trait.

The high value placed on having a positive attitude among older people with MS suggests that tailored interventions using positive psychology may help to maintain and foster positive thinking in people who have been recently diagnosed with MS. Positive psychology encourages the understanding of psychosocial strengths and resources rather than focusing on deficits and vulnerabilities [45]. Recently, the feasibility of positive psychology to help manage chronic conditions including MS has gained some attention [45–47]; however more work is required to provide meaningful direction to clinicians and those living with MS seeking to develop long-term self-management plans.

#### 3.8.3. Creating Supportive Environments for a Healthy Lifestyle

People of all ages are reminded through public health messaging about lifestyle choices and habits that were identified by older people with MS as being most important to healthy aging with MS. Previous analysis of this older group of people with MS suggests that, despite moderate disability, they live healthier lifestyles compared to other people of their age [2]. They exercise more, drink less alcohol, and abstain from smoking. Therefore, self-management strategies seeking to foster these behaviors among young and middle age people with MS need to go beyond education about the benefits of adopting healthy behaviors. Rather, young and middle aged people with MS planning for aging in place should identify resources in their communities that allow them to engage in and remain engaged in healthy lifestyle choices and habits as their abilities change. Community organizations and health providers must offer options for people with disabilities [48–50]. Accessible exercise facilities, affordable healthy food choices, flexible work environments, and inclusive community activities need to be a priority among policy makers and the greater community in order to foster healthy aging not only for people with MS but also for people at any age and regardless of health status.

## 4. Limitations

Although this study provides important insights about healthy aging with MS, there are some limitations to consider. First, an open ended survey question is a relatively crude qualitative tool compared to interviews; because of this the data may sometimes lack depth limiting the researchers' ability to fully understand the phenomena. This may have been somewhat mitigated by the large breadth of data that was available to the researchers. Next, the sample consists of community dwelling older individuals with MS so those living in supportive care environments are underrepresented. Furthermore, those with reading and writing problems may be underrepresented. As with most survey-based studies, people with more formal education and a positive outlook may be more likely to participate [51] which may affect the generalizability of findings. The survey also did not address possible cognitive impairment in participants which may have impacted results in terms of recall bias. Also, the respondents are living in a Western country with a relatively well-funded public health care system so their perceptions of healthy aging may not apply to those living in dissimilar environments. Future research may seek to better understand healthy aging with MS from individuals in developing countries. There were only 51 participants in the oldest old group which may point to a ‘survivor bias.' Although it could be argued that the oldest group may be those with a milder form of MS, the disability scores on the Barthel Index suggest otherwise.

## 5. Conclusions

Learning from the experiences of older adults who have lived for decades with MS can help young and middle aged people with MS plan to age in their own homes and communities. Our data suggests that older people with MS clearly prioritize specific factors which contribute to healthy aging. Social connections, attitude and outlook on life, and healthy lifestyle choices and habits represent modifiable factors that can be positively impacted through targeted self-management strategies. Older people successfully aging in their own homes seem to have a positive outlook and engage in healthy lifestyle behaviors.

## Figures and Tables

**Table 1:** Participant characteristics by age group.

	55–64	65–74	75+	*p* value
*N*	377	255	51	—
Age (mean, SD)	60 (2.51)	69 (2.71)	79 (3.57)	—
Gender (female, male)	302, 75	190, 65	39, 12	0.274
Years of education (mean, SD)	13.62 (2.38)	13.38 (2.76)	13.25 (2.91)	0.397
Years with symptoms (mean, SD)	30.68 (7.5)	34.76 (10.06)	40 (13.25)	0.0001^*∗*^
Mental health status (HADS, range 0–42)	12.11 (6.6)	11.62 (6.1)	11.32 (5.87)	0.517
Level of disability (Barthel Index, range 0–100)	78.47 (24.04)	75.33 (24.39)	70.49 (24.36)	0.460
Living with spouse/partner (%)	70.3%	73.3%	58.8%	0.114
Living in own home (%)	97.3%	94.9%	94.1%	0.209

^*∗*^Significant at <0.05.
